# Evolution of Concepts Regarding the Diagnostic and Prognostic Significance of Glial Fibrillary Acidic Protein (GFAP)-Positive Extracellular Vesicles

**DOI:** 10.3390/biomedicines14051116

**Published:** 2026-05-14

**Authors:** Natalia Yunusova, Dmitry Svarovsky, Polina Panfilova, Anastasia Ryabova, Evgeniya Kaigorodova, Evgeniya Sidenko, Polina Gervas, Aleksey Molokov, Irina Kondakova

**Affiliations:** 1 Cancer Research Institute, Tomsk National Research Medical Center of the Russian Academy of Sciences, 5, Kooperativny str., 634009 Tomsk, Russia; svarovsky.d.a@gmail.com (D.S.); ranigor@mail.ru (A.R.); zlobinae@mail.ru (E.K.); sidenkoevgeniyaaleksandrovna@gmail.com (E.S.); pgervas@yandex.ru (P.G.); a.y.molokov@gmail.com (A.M.); kondakova@oncology.tomsk.ru (I.K.); 2 Department of Biochemistry and Molecular Biology with the Course of Clinical Laboratory Diagnostics, Siberian State Medical University, 2, Moskovsky Tract, 634050 Tomsk, Russia; 3 Clinical and Diagnostic Laboratory, Medical Association Family Medicine Center, 22B, Trifonova str., 634050 Tomsk, Russia; teofen@yandex.ru

**Keywords:** GFAP, extracellular vesicles, exosomes, protein corona, astrocyte-derived extracellular vesicles, Schwann cells, traumatic brain injury, Alzheimer’s disease, stroke, glioblastoma multiforme

## Abstract

This review demonstrates that the diagnostic and prognostic significance of glial fibrillary acidic protein (GFAP) is not limited to its use as a marker of astrocytic damage but should also be considered in the context of the diversity of GFAP isoforms, their heterogeneous tissue-specific expression and their pronounced association with extracellular vesicles (EVs). The data presented in this review indicate that GFAP-positive (GFAP+) EVs possess broad clinical relevance in both acute and chronic pathologies of the nervous system, including ischemic stroke, traumatic brain injury, glioblastoma, and potentially diabetic and drug-induced polyneuropathy. Particular attention is given to the critical analysis of methodological approaches for studying GFAP+ EVs, including discussion of their proposed biogenesis, mechanisms of intravesicular incorporation of cytoskeletal fragments, and the hypothetical sorption of GFAP within the vesicular protein corona. A principal conclusion of this work is that, despite the high translational potential of GFAP+ vesicles as a novel liquid biopsy platform, further implementation of this approach in clinical practice will require standardization of EV isolation protocols, harmonization of phenotyping methodologies in accordance with MISEV 2023 recommendations, and large-scale prospective studies aimed at validating the biological nature, origin, and clinical reproducibility of identified GFAP-associated vesicular subpopulations.

## 1. Introduction and Review Methodology

Glial fibrillary acidic protein (GFAP) is a member of the intermediate filament type III family of proteins, along with desmin (expressed in skeletal and cardiac muscle cells), vimentin (expressed in several cell types, including glial cells), and peripherin (expressed in neurons) [1]. GFAP is primarily found in the central nervous system (CNS) in immature and mature astrocytes, but in addition to the CNS, GFAP is expressed in non-myelinating Schwann cells (SCs) and satellite glial cells (SGCs) of the peripheral nervous system (PNS). It is believed that GFAP is not typically secreted under normal conditions. Instead, it is released into the cerebrospinal fluid and bloodstream following astrocyte death, injury, or neuroinflammation. Soluble GFAP as a diagnostic and prognostic biomarker for brain injury is widely reported in the literature [2,3]. In the present review, we focused on a critical analysis of the literature data on the prospects of GFAP-positive (GFAP+) extracellular vesicles (EVs) as diagnostic and prognostic markers of nervous system pathology.

This approach is used due to the complex and fundamentally different biogenesis of small and medium/large EVs, their stability in circulation, and the presence of a protein corona (PC) in absolutely all EVs circulating in the blood, the composition of which can reflect the sorption of freely circulating GFAP onto EVs in blood plasma. Compared with the quantitative assessment of freely circulating GFAP, the detection of GFAP+ EVs and the analysis of their cargo open a new window of opportunity for the search for informative biomarkers. This analytical review discusses three biologically distinct subjects: freely circulating GFAP, intravesicular GFAP cargo, and GFAP found on or around EVs as part of the PC of the EV. To distinguish intravesicular GFAP from PC-associated GFAP, protease protection assays combined with membrane-disrupting agents can be applied. Protease treatment alone degrades externally bound (corona) proteins, whereas intraluminal GFAP remains protected unless vesicle membranes are permeabilized (e.g., by detergents). Additional confirmation may be obtained using immuno-electron microscopy or differential detergent-based fractionation approaches.

In this review, we used the MISEV 2023 guidelines for characterizing vesicle types according to (a) physical characteristics such as size (small EVs < 200 nm and medium/large vesicles from 200 to 2000 nm) or density; (b) biochemical composition (e.g., CD9+ EVs and GFAP+ EVs); and (c) descriptions of the conditions of EV production or their cellular origin (e.g., hypoxic EVs) [4]. Small EVs should not be called exosomes unless their endosomal origin is proven according to MISEV 2023 recommendations. Consensus regarding specific markers of EV subtypes, such as “exosomes” of endosomal origin and “ectosomes” (microparticles/microvesicles) derived from the plasma membrane, has not yet been reached. However, many researchers continue to use the terms “exosomes,” “microvesicles,” and “microparticles”, so we tried to adapt the available literature to the MISEV 2023 recommendations.

This narrative review was conducted through a structured literature search of the PubMed/MEDLINE and Google Scholar databases to identify relevant studies addressing the biology, diagnostic significance, and translational potential of GFAP+ EVs. Search terms included combinations of the following keywords: “GFAP”, “glial fibrillary acidic protein”, “extracellular vesicles”, “exosomes”, “astrocyte-derived extracellular vesicles”, “Schwann cells”, “satellite glial cells”, “traumatic brain injury”, “Alzheimer’s disease”, “stroke”, “glioblastoma”, “glioblastoma multiforme”, “diabetic neuropathy”, “drug-induced neuropathy”, and “protein corona”. The search was limited to English-language publications. No strict date restriction was applied for seminal studies related to GFAP discovery, molecular structure, isoform characterization, and fundamental aspects of extracellular vesicle biology. For translational, clinical, and mechanistic studies specifically evaluating circulating GFAP-positive extracellular vesicles, emphasis was placed on the literature published between 2010 and 2026.

Additional relevant references were identified through manual screening of bibliographies. Articles lacking direct relevance to GFAP biology or EV research, non-peer-reviewed sources, conference abstracts without full texts, and publications with insufficient methodological description were excluded. Priority was given to original experimental studies, translational clinical investigations, systematic reviews, meta-analyses, and methodological consensus papers. In cases where contradictory or methodologically heterogeneous findings were identified, they were critically discussed with attention to technical limitations, cohort size, analytical platform variability, and uncertainty regarding vesicular versus non-vesicular protein localization.

## 2. Structure and Function of GFAP

GFAP discovery was reported in the fall of 1969 at a meeting of the International Society of Neurochemistry by Lawrence Eng, who described GFAP in a study of proteins in three different samples of glial scars in the CNS obtained from patients with multiple sclerosis, postoperative scars, and hydrocephalus [1]. GFAP shares a common structure with all intermediate filaments: two globular domains at the N- and C-termini, which are connected by a rod-shaped domain consisting of α-helices. The N-terminal head domain is critical for filament assembly, the rod domain plays a major role in dimer formation through polypeptide twisting, and the C-terminal tail domain is important for intermediate filament stabilization [5]. Like many other intermediate filament proteins, GFAP monomers form dimers by interactions between two rod-shaped domains that form a double supercoiled helix [6]. Dimers are the basic structural unit of intermediate filaments [7]. Cytoplasmic intermediate filaments form non-polar strands called tetramers, which are composed of two dimers with antiparallel orientation. Several strands of tetramers further combine to form octamers, etc., until a mature intermediate filament strand with an average diameter of 10 nm is formed. The gene encoding human *GFAP* consists of nine exons and is located on chromosome 17 (17q21.1-q25). Currently, six GFAP isoforms have been described: GFAP-α, GFAP-β, GFAP-γ, GFAP-δ/ε, GFAP-κ, and GFAP-ζ. GFAP-α, a 432-amino acid protein found in the brain, spinal cord, and PNS, is the predominant isoform in the human body. It is the subject of most publications and has the most studied clinical significance [8]. The GFAP-β and GFAP-γ isoforms are produced by RNA reading from alternative transcription start sites and, accordingly, have alternative N-terminal domains [7], while the GFAP-δ/ε, GFAP-κ, and GFAP-ζ isoforms with a variable C-terminal domain are produced by alternative splicing, a process unique to GFAP among cytoplasmic intermediate filaments other than synemin (which belongs to type IV intermediate filaments) [6]. GFAP-β is highly expressed in non-myelinating SCs in the PNS [9], and GFAP-γ mRNA is abundant in the corpus callosum of the human brain and is also present in the bone marrow and spleen of mice [10]. GFAP-δ/ε is predominantly expressed by astrocytes in the subventricular zone of the brain. The expression of the GFAP-κ isoform has been described in the spinal cord, brain, and human brain, and the expression of the GFAP-ζ isoform is limited to the brain [11]. Four additional splice variants, GFAPΔEx6, GFAPΔ164, GFAPΔ135, and GFAPΔEx7, have been detected in some astrocytes throughout the brain. Collectively, these isoforms are referred to as GFAP+1, reflecting their formation by a single-nucleotide frameshift. In Alzheimer’s disease, GFAP+1 is expressed only in a subset of astrocytes with long processes, the number of which increases as the disease progresses [11,12]. Immunohistochemical studies also showed an increase in the number of GFAPΔEx6- and GFAPΔ164-positive astrocytes in focal brain lesions in chronic epilepsy [13]. In addition, specific GFAP-expressing splenic cell lines have been described, although this has been demonstrated only in experimental models [14].

Table 1 shows cells that predominantly contain different GFAP isoforms.

**Figure 1:**
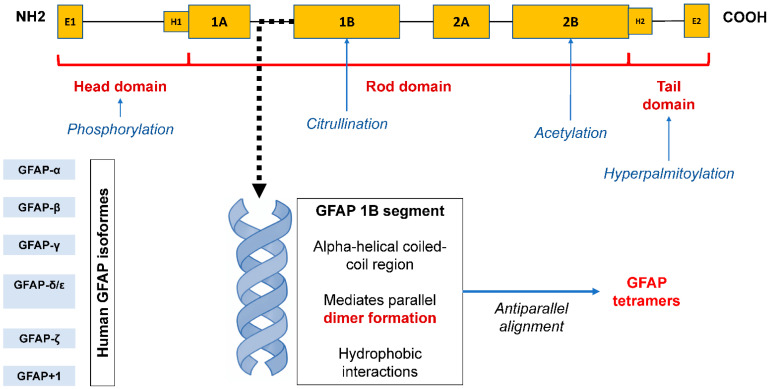
Structural organization of GFAP and the role of the 1B domain in intermediate filament assembly. Note: GFAP consists of an N-terminal head domain, a central α-helical rod domain (subdomains 1A, 1B, 2A, and 2B), and a C-terminal tail domain. The head domain is involved in filament initiation and is subject to phosphorylation, whereas the rod domain mediates dimerization and higher-order assembly. The 1B segment represents an α-helical coiled-coil region that facilitates parallel dimer formation through hydrophobic interactions. These dimers subsequently associate in an antiparallel manner to form non-polar tetramers, which serve as fundamental building blocks for intermediate filament formation. Post-translational modifications, including citrullination, acetylation, and hyperpalmitoylation, may regulate GFAP structure and function. The scheme represents a conceptual model based on known structural features of type III intermediate filaments.

A diagram illustrating the structure of the GFAP protein domains is shown in Figure 1.

Currently, standard methods for GFAP detection (ELISA and ultrasensitive platforms) are primarily focused on the identification of the predominant GFAP-α isoform and do not distinguish rare variants [8,11]. Differentiation of GFAP isoforms in the blood is theoretically possible using specialized approaches such as mass spectrometry or immunoassays employing antibodies against unique epitopes. Plasma GFAP levels reliably reflect the degree of reactive astrocytic proliferation and, therefore, dynamically respond to pathological conditions. GFAP has several advantages over other neuron-specific biomarkers, such as neurofilament light chain (NfL) and Tau. For example, in Parkinson’s disease, plasma GFAP levels in patients with mild cognitive impairment were shown to predict conversion to dementia (AUC = 0.90), outperforming NfL and Tau [16]. While NfL and Tau, as neuronal markers, primarily reflect axonal and neuronal cell body damage, GFAP is largely specific for glial (astrocytic) injury. Thus, GFAP complements other biomarkers by providing a dynamic reflection of neuroinflammation and astrogliosis.

Regarding the functions of GFAP, its role in the CNS has been well studied. GFAP is the primary structural component of the astrocyte cytoskeleton. It provides mechanical strength and shape to astrocytes, supports neighboring neurons, and regulates the blood–brain barrier (BBB). It is important to note that GFAP and vimentin are key components responsible for the assembly and elongation of intermediate filaments in astrocytic processes. The GFAP network has also been established to regulate intracellular vesicle motility [15] and chaperone-mediated autophagy [17,18]. Studies on *GFAP*-knockout animals showed that *GFAP-null* mice and rats exhibited normal development, with vimentin providing compensatory structural support in glial cells, ensuring normal birth and reproduction. However, the absence of GFAP impaired neural tissue repair, leading to deficient CNS/PNS injury response [19], impaired SCs differentiation, slowed sciatic nerve regeneration [20], and reduced reactivity to injury [8,21].

## 3. Expression, Secretion, and Study of Serum/Plasma GFAP as Diagnostic and Prognostic Marker of CNS Pathology

GFAP is primarily found in the CNS in immature and mature astrocytes of the gray and white matter of the brain and spinal cord [22]. In addition to the CNS, GFAP is expressed in non-myelinating SCs and SGCs of the PNS, Müller cells of the retina, and in intestinal glial cells [23,24]. It is expressed in liver and pancreas stellate cells, Leydig and Sertoli cells [25], lens epithelial cells, chondrocytes, and osteocytes [24]. STAT3 and AP-1 are key transcription factors regulating *GFAP* gene expression. STAT3 is crucial for the initiation of protein synthesis during active cell division during development and for the regulation of protein expression at rest. AP-1 is primarily responsible for enhancing *GFAP* expression during reactive gliosis following injury [26,27]. Growth factors, such as NGF, FGF, and TGFβ, can activate *GFAP* gene transcription, leading to increased GFAP levels. Some hormones (thyroid hormones and glucocorticoids) can also activate *GFAP* transcription. The effect of thyroid hormones may be mediated by activation of the ROCK signaling pathway. These hormone and growth factor-based *GFAP* gene regulators are potentially important for the induction of mature astroglia formation [10]. Increased GFAP levels were found during reactive gliosis following injury, neurodegeneration, and in tumors like glioblastoma [2,3,28,29,30]. Since GFAP is the main structural framework of astrocytes, damaged cells release proteolyzed GFAP degradation products (38–44 kDa) and intact protein (50 kDa) into the environment, such as the interstitial fluid, after which they enter the subarachnoid space and then the general circulation via direct venous drainage or diffuse through the damaged BBB [31,32].

Numerous prospective cohort or multicenter observational studies have confirmed that serum or plasma GFAP levels are promising biomarkers of traumatic brain injury (TBI). However, the diagnostic accuracy of GFAP is significantly affected by age. The TRACK-TBI pilot study found that in a subgroup of 169 patients with mild TBI, the ability of GFAP to identify computer tomography (CT)-positive intracranial injuries decreased with increasing age (with an AUC of 0.73 in patients aged > 60 years compared with an AUC of 0.93 in patients aged < 40 years). Other glial biomarkers (e.g., S100B) have reduced specificity in older adults compared with younger adults. This age effect may result from incipient neurodegeneration, differences in anatomical location, and types of injury in older adults. Importantly, in TBI, blood GFAP levels are sensitive to subclinical intracranial pathologies that are not visualized on initial head CT [2]. In 243 participants with moderate-to-severe TBI, adding blood GFAP and microtubule-associated protein 2 measurements to known clinical predictors (age, gender, and the Glasgow Severe Infarction Scale) improved the prediction of a favorable 6-month outcome compared with clinical assessment alone [33].

Recent findings confirm that GFAP is a valuable prognostic tool in stroke patients, although an important limitation of the diagnostic use of blood GFAP may be its low specificity for differentiating stroke subtypes [2]. With the exception of studies on the role of serum/plasma GFAP in TBI, most studies discussed in the reviews by Abdelhak A. et al. (2022) [2] and Zheng X. et al. (2024) [3] (on brain tumors, multiple sclerosis, neurodegenerative diseases, psychiatric diseases, and systemic diseases) were single-center, retrospective, or had methodological limitations (a small sample size). The lack of direct comparability between studies measuring blood GFAP is a major challenge, driven by significant methodological heterogeneity, differing assay platforms, and a lack of standardized values [2,3]. Therefore, the value of circulating GFAP as a diagnostic and prognostic marker in these pathologies has not yet been clearly defined.

## 4. GFAP-Positive Vesicles as Marker of CNS Pathology

Investigation of GFAP within EVs (specifically astrocyte-derived EVs (ADEVs)) offers a promising alternative to measuring total free GFAP levels in the blood. Exosomes are small (30–150 nm) EVs formed by the inward budding of intraluminal vesicles, which subsequently fuse with the outer membrane to release these vesicles. Their composition depends on the parent cell. Oligodendrocyte exosomes contain myelin proteins that support oligodendroglial function, while microglial exosomes contain immune-related proteins, reflecting their innate immune function in the CNS [34,35]. GFAP-positive EVs derived from astrocytes increase in concentration during astrocyte activation and in experimental autoimmune encephalomyelitis models [36]. Astrocytes are critical factors of ischemic injury, neuroinflammation, and immune-mediated inflammation. Their functions are regulated by interactions with other types of CNS cells through EVs. GFAP is a classic marker of astrocytes, which constitute up to 40% of all cells in the CNS. Extensive studies indicate that GFAP+ EVs are promising biomarkers for Alzheimer’s disease [37], schizophrenia [38], stress-induced exhaustion disorder [39], acute ischemic stroke, TBI, and glioblastoma multiforme [40,41,42,43,44]. The potential role of astrocyte-secreted GFAP-EV load in response to cerebral ischemia was assessed in the study by T. Forró et al. (2024) [40]. The levels of GFAP+ EVs were increased in the blood on days 1 (*p* = 0.007) and 7 (*p* = 0.019) following ischemic stroke, but not at 1 month (*p* = 0.344), compared with controls. A positive correlation was observed between the modified Rankin scale and the GFAP level in astrocytic EVs on days 1 and 7 after ischemic stroke (r = 0.58; *p* = 0.010) and (r = 0.57; *p* = 0.013), respectively. According to research, the level of full-length GFAP protein (50 kDa) in ADEVs isolated from plasma via immunoprecipitation and analyzed by Western blotting acts as a dynamic biomarker for acute ischemic stroke [40]. Currently, the very small number of studies, limited cohort sizes, different approaches to vesicle isolation, and different vesicle pellet enrichment strategies make comparison of study results difficult and make clinical interpretation of results extremely cautious (Table 2).

Most studies evaluating the sensitivity and specificity of GFAP+EVs versus free circulating GFAP have concentrated on CNS disorders and brain tumors. For example, in TBI, EV-associated GFAP at early time points correlates with injury severity and CT-detected damage; however, plasma GFAP demonstrates comparable performance [41]. In the same study, EV-GFAP levels showed a significant increase (approximately 2.8-fold in CT-positive cases), but free GFAP levels were also elevated (≈3.4-fold), suggesting that the isolation of vesicular GFAP does not yet provide a substantial gain in sensitivity [41].

**Figure 2:**
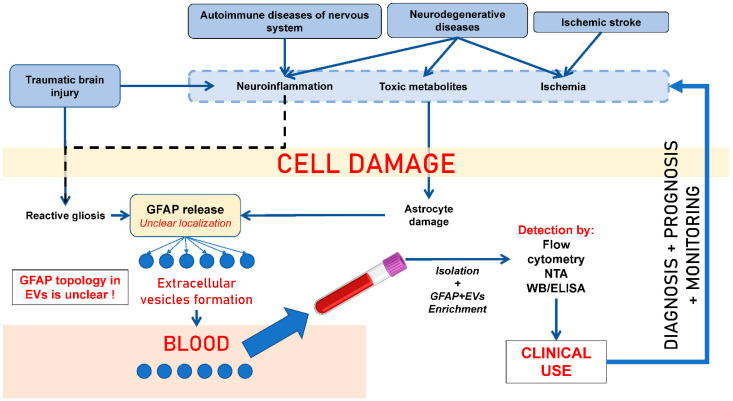
Pathophysiological mechanisms leading to the release of GFAP+ EVs in neurological disorders. Note: GFAP—glial fibrillary acidic protein; EVs—extracellular vesicles; WB—Western blotting; NTA—nanoparticle tracking analysis; ELISA—enzyme-linked immunosorbent assay; ADEVs—astrocyte-derived EVs.

GFAP+ EVs are actively studied as markers of glial tumors, primarily in patients with glioblastoma multiforme. Recurrent glioblastomas are typically characterized by diffuse, pronounced staining for GFAP; therefore, GFAP+ EVs may be present in high quantities in glioblastoma patients’ blood plasma [30,36,43,44,45,46,47]. GFAP+ EVs are used to monitor anti-relapse therapy in patients with gliomas. CD9+/GFAP+/Survivin+ and CD9+/Survivin+ EVs are present in the circulation of patients with gliomas, and a sustained reduction in their numbers after anti-survivin immunotherapy may be associated with longer progression-free survival. Thus, the detection of GFAP+ EVs in blood plasma may be useful for monitoring tumor response in patients with malignant gliomas [44]. A research group from Guangzhou Medical University (China) has developed a multicellular 3D co-culture model to study the interaction of macrophages, multipotent mesenchymal stromal cells, tumor cells, and EVs in glioblastoma multiforme. All three cell types have been shown to interact both directly and through paracrine signals, and EVs secreted in these structures mediate these interactions through the internalization and transfer of microRNA, suppressing the growth, migration, and invasiveness of tumor cells (the levels of Ki67 and GFAP in the cells were also assessed) [48]. A similar study was conducted by Gudbergsson J. M. et al. (2019) [49]. They tested a tumorsphere model of glioblastoma multiforme. Intercellular heterogeneity in tumorspheres was investigated using immunofluorescence staining of nestin/vimentin and GFAP in cells and EVs, which revealed that nestin and vimentin were highly expressed at the tumorsphere periphery, while GFAP was predominantly expressed in cells in the tumorsphere core. The authors also showed that this phenotypic gradient was present in vivo after implantation of dissociated glioblastoma tumorspheres, with cells migrating from the tumor being nestin-positive and GFAP-negative. The authors concluded that these models were relevant as a preclinical platform for assessing cell migration in tumors and screening for drug efficacy [49]. The putative role of circulating GFAP+ EVs in the development of CNS pathology is shown in Figure 2.

Analysis of exosome secretion from primary glial cultures using flow cytometry with antibodies against CD63, GFAP and Tsg101 revealed an increased number of exosomes after stimulation of cells with IL-1β. This proves the feasibility of secreting “true” GFAP+ EVs and detecting such EVs via high-throughput flow cytometry [36]. It is assumed that GFAP is also present in the PC of EVs of non-astrocytic origin. The PC is formed not during intracellular biogenesis, but through the adsorption of proteins from biofluids (plasma and interstitial fluid) onto the EV surface after secretion. The PC consists of endogenous ligands that can mask the membrane composition of EVs and block cell membrane receptors, thus preventing internalization. In addition, the PC of EVs creates new opportunities for diagnosing various diseases due to the fact that its composition depends on many physical and chemical characteristics of the EVs themselves and the environment, including the concentration of biomolecules. The more biomolecules in the environment, the higher the probability of their adsorption in the PC [43,50]. Therefore, it is logical to assume that elevated free GFAP in the blood during neurological pathologies is accompanied by increased GFAP on circulating EVs. However, the concentration of free protein in the EV microenvironment is apparently not the only factor influencing its sorption into PC vesicles. The process of PC formation is currently being intensively studied, and our hypothesis regarding the presence of GFAP in the corona of non-astrocytic-origin EVs is currently a scientific hypothesis that requires confirmation.

## 5. GFAP-Positive Vesicles as Marker of PNS Pathology

Beyond the CNS, GFAP is expressed in PNS by non-myelinating SCs, SGCs, enteric glia, and hepatic stellate cells [7,8,51,52]. It is known that astrocytes are a major glial cell type in the human brain, with the adult human neocortex containing approximately 4.8 to 7.8 billion astrocytes, generally comprising 20–40% of the total glial cell population. They are roughly equinumerous to or slightly outnumber neurons, depending on the brain region, with their numbers correlating with brain size. Meanwhile, non-myelinating SCs constitute a major SC population in the PNS, frequently outnumbering myelinating cells in cutaneous nerves. Specifically, precise numerical data for the total count of non-myelinating SCs in the entire human body is not readily available in the literature, but they represent a significant portion of the total PNS glial population [6,8,52]. Thus, both cell populations are dominant in the human CNS and PNS, respectively. It can be hypothesized that GFAP+ vesicles produced by both astrocytes and non-myelinating SCs may be equally present in the circulation. The concentration of these vesicles likely reflects both the acute injury and the functional stimulation of these cells. The lack of studies in clinical cohorts currently allows this position to be formulated only as a scientific hypothesis, which requires further research.

Nevertheless, circulating GFAP+ vesicles as possible markers of PNS pathology should be discussed primarily in the context of the most common variants of peripheral polyneuropathy-diabetic (DNP) and drug-induced (DINP) neuropathies, which differ in etiology but share similar final-stage damage. It is believed that hyperglycemia is the leading factor in nerve tissue damage in diabetes mellitus. Intracellular hyperglycemia reduces the activity of sorbitol dehydrogenase. Sorbitol, being a hexatomic alcohol, accumulates in the bodies of neurons, SCs, the endothelium and nerve processes, which can cause disturbances in osmotic homeostasis, with subsequent cell damage [53,54]. Hyperglycemia accelerates non-enzymatic and enzymatic glycation of structural proteins of the nerve fiber (myelin and tubulin), forming advanced glycation end-products (AGEs) that are highly exacerbated by fructose. These AGEs disrupt nerve function by impairing neuronal metabolism, axonal transport, nerve impulse conduction, and the regenerative capacity of Schwann cells and stimulate the synthesis of proinflammatory cytokines [54]. EVs were shown to be effectively internalized by endocytosis and micropinocytosis mechanisms by neurocytes and glial cells, carry markers of insulin resistance, and functionally active proteins (receptors, cytokines, and enzymes), and can participate in the pathogenesis of DNP [54].

General mechanisms of DINP development due to the use of antitumor drugs (taxanes, platinum drugs, and vinca alkaloids) are well known. In contrast with the CNS, peripheral axons are not protected by the BBB, allowing cytostatic metabolites to penetrate into nerve fibers by direct diffusion and accumulate in them, causing their damage. The molecular mechanisms of DINP include impaired microtubule dynamics and axonal transport, axon demyelination, impaired actin remodeling in PNS cells, oxidative stress, endothelial damage with subsequently impaired PNS cells, induction of apoptosis, mitochondrial dysfunction, and proteolytic stress. It is believed that PNS neurons suffer indirectly due to the predominant damage to SCs [55,56,57]. This may also be reflected through the secretion of GFAP+ EVs by non-myelinating SCs, and these changes may be reflected quantitatively and qualitatively through the GFAP+ EVs’ cargo in the blood plasma.

It was shown that the composition of microRNAs and proteins of circulating exosomes changed significantly in both DNP and DINP. EVs of blood plasma from healthy rats were enriched in MiR-20b-3p compared with exosomes from diabetic rats. Intravenous administration of MiR-20b-3p-enriched exosomes to diabetic rats ameliorated the severity of DNP in functional tests. Histological examination revealed sciatic myelin regeneration, increased intraepidermal nerve fibers, distal local blood perfusion, and enhanced neuromuscular junction and muscle spindle innervation after exome administration from healthy rat plasma. MiR-20b-3p was shown to regulate SCs’ autophagy by targeting STAT3 and thereby inhibiting DNP progression [58]. Intravenous administration of SC-derived EVs (SC-EVs) to type 2 diabetes mellitus db/db mice with DNP improved sciatic nerve conduction velocity as well as thermal and mechanical sensitivity. These functional improvements were associated with an increase in epidermal nerve fibers and sciatic nerve remyelination. RT-PCR and Western blot analysis of sciatic nerve tissues showed that SC-EV treatment normalized miR-21, -27a and -146a, semaphorin 6A, Ras, RhoA, PTEN and NF-κB levels. In vitro data demonstrated that SC-EVs promoted neurite outgrowth of diabetic dorsal root ganglion (DRG) neurons and SC migration when exposed to high glucose [59].

You M. et al. (2023) [60] found that SC-EVs were able to alleviate the loss of mechanical nociceptive sensitivity due to DINP in rats in vivo. Histology showed that SC-EVs attenuated anticancer drug-induced loss of plantar intraepidermal nerve fibers and DRG neuron damage. SC-EVs alleviate DINP through the miR-21-mediated PTEN signaling pathway [60]. GFAP+ SGCs in the DRG were thought to influence the function of sensory neurons through intercellular communication via EV secretion. After oxaliplatin treatment, an increase in GFAP immunoreactivity was detected in SGCs in vitro. Oxaliplatin treatment stimulated the secretion of SGC-EVs, which were efficiently internalized by neurons isolated from the DRG when co-incubated. Moreover, after incubation with conditioned SGC-EVs (after treatment with 4 μM of oxaliplatin), the percentage of neurons overexpressing reactive oxygen species increased. SGC-EVs treated with oxaliplatin in vitro exerted a pronociceptive effect on DRG neurons and induced mechanical hypersensitivity in naive mice, possibly via their miRNA cargo (miR-324-3p, miR-181a-5p, and miR-122-5p) [61].

**Figure 3:**
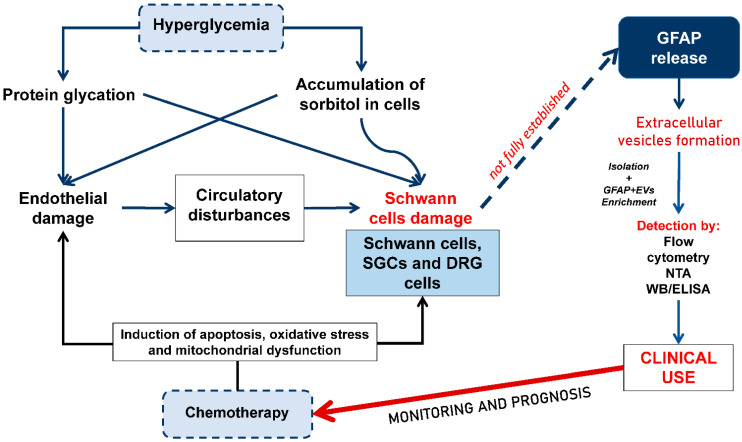
Proposed mechanisms of GFAP+ EV formation under hyperglycemia and chemotherapy-induced cellular stress. Note: GFAP—glial fibrillary acidic protein; EVs—extracellular vesicles; DRG—dorsal root ganglia; SGCs—satellite glial cells; WB—Western blotting, NTA—nanoparticle tracking analysis; ELISA—enzyme-linked immunosorbent assay.

The putative role of circulating GFAP+ EVs in the development of DNP and DINP is shown in Figure 3. We believe that in peripheral nervous system injury (e.g., DNP), elevated levels of GFAP+ EVs may originate from SCs or other peripheral sources of GFAP. Even if GFAP isoforms cannot be differentiated in plasma, their presence may be explained by peripheral GFAP expression: for instance, GFAP-β is described as the predominant isoform in non-myelinating SCs (Table 1), and GFAP is known to be expressed in human SCs. Therefore, an increase in GFAP+ EVs in PNS pathology is biologically plausible, reflecting damage to glial components of peripheral nerves rather than exclusively CNS injury.

## 6. Methodological Aspects of the Study of GFAP-Positive Extracellular Vesicles

The lack of standardized protocols for vesicle isolation (both the total fraction and tissue-specific or cell-specific vesicle fractions), which leads to significant variability in GFAP+ EV quantification, remains the major challenge in biomarker research. It is important to note that, according to the MISEV 2023 recommendation, various EV isolation methods can be used (differential centrifugation, ultracentrifugation with ultrafiltration, approaches using commercial immunoprecipitation-based kits, size-exclusion chromatography, fluorescence-activated sorting, etc.). The key position of MISEV 2023 is that the isolation procedure must be described in as much detail as possible, as well as the rationale for the choice of approaches for EV isolation [1,2]. It is also necessary to characterize the isolated vesicles in detail using a combination of methods (electron or cryo-electron microscopy, nanoparticle tracking analysis (NTA), dynamic light-scattering analysis, and evidence of the presence of tetraspanins on the surfaces of isolated vesicles) [4].

The precise topology of GFAP within EVs is a subject of debate. Traditionally, GFAP is considered a cytoplasmic protein that enters vesicles during their biogenesis (e.g., during the formation of multivesicular bodies) or through the “capture” of cytoskeletal fragments during biogenesis by medium/large EVs. Published studies of this type are currently extremely limited, and the approaches used in these studies (immunoprecipitation with Western blotting and various flow cytometry techniques) do not allow a definitive conclusion to be drawn regarding whether GFAP is exclusively located within astrocytic vesicles, whether it is released onto the EV membrane during the biogenesis and secretion of small or large EVs, or whether GFAP is localized exclusively within the corona of GFAP+ EVs [36,38,40,43].

The study by Tóth EÁ (2021) [50] demonstrated that EVs carry a diffuse (patchy) PC, consistent with prior expectations. It was further shown that large protein aggregates present in blood plasma are also associated with the EV surface. This finding provides a straightforward explanation for the observed high proteomic overlap between corona-coated EVs and plasma protein aggregates. Immunoelectron microscopy has revealed corona proteins—primarily ApoA1, ApoB, ApoC3, ApoE, complement factors C3 and C4B, fibrinogen α-chain, immunoglobulin light and heavy chains, and albumin—co-localizing with CD63 around EVs. In this and other studies, no clear evidence has been presented for the presence of GFAP within the EV PC [50]. According to data from the ExoCarta and Vesiclepedia databases, GFAP is consistently reported at the level of detection in EV-enriched fractions, most commonly via mass spectrometry (MS), occasionally supported by general EV validation workflows (e.g., electron microscopy, NTA, and canonical EV markers such as CD63, TSG101, or ALIX). However, none of the analyzed records provide direct, GFAP-specific evidence resolving its spatial localization with respect to the vesicle.

In particular, there is no experimental evidence demonstrating that GFAP is localized within the EV corona (i.e., adsorbed to the vesicle surface), nor within the vesicle lumen or membrane. Critically, the datasets lack localization-resolving approaches such as immunogold electron microscopy targeting GFAP, protease-based treatment with and without membrane disruption, or biochemical fractionation strategies designed to distinguish surface-associated proteins from intravesicular cargo.

As a result, the presence of GFAP in these datasets should be interpreted conservatively: it reflects its association with EV preparations but does not establish whether GFAP represents a bona fide vesicular component (luminal or membrane-associated), a corona protein-acquired post-secretion, or a co-isolated contaminant derived from cellular debris or non-vesicular particles. This ambiguity is further compounded by the known propensity of intermediate filament proteins, including GFAP, to appear in proteomic datasets of EV fractions under certain isolation conditions (Table 3).

## 7. Conclusions

This review demonstrates that the diagnostic and prognostic significance of GFAP is not limited to its use as a marker of astrocytic damage but should be considered in the context of the diversity of GFAP isoforms, their heterogeneous tissue-specific expression and pronounced association with EVs. The data presented in this review indicate that GFAP+ EVs possess broad clinical relevance in both acute and chronic pathologies of the nervous system, including ischemic stroke, TBI, glioblastoma, and potentially diabetic and drug-induced polyneuropathy. In the latter context, GFAP+ vesicles may reflect not only the degree of damage to astrocytes, SCs, and SGCs, but also their intercellular communication within the tissue microenvironment. Particular attention is given to the critical analysis of methodological approaches for studying GFAP+ EVs, including discussion of their proposed biogenesis, mechanisms of intravesicular incorporation of cytoskeletal fragments, and the hypothetical sorption of GFAP within the vesicular PC. This review substantially expands beyond the limitations of conventional descriptive analyses of experimental studies and EV databases such as ExoCarta and Vesiclepedia, which confirm only the presence of GFAP in EV-associated fractions but do not allow reliable determination of its precise subvesicular localization. A principal conclusion of this work is that despite the high translational potential of GFAP+ vesicles as a novel liquid biopsy platform, further implementation of this approach in clinical practice will require standardization of EV isolation protocols, harmonization of phenotyping methodologies in accordance with MISEV 2023 recommendations, and large-scale prospective studies aimed at validating the biological nature, origin, and clinical reproducibility of identified GFAP-associated vesicular subpopulations.

## Figures and Tables

**Table 1:** GFAP isoforms in cells and tissues.

GFAP Isoforms		Number of Amino Acids	Localization	References
GFAP-α		432	- Predominant isoform in human astrocytes - Human non-myelinating SCs	[5,8,9]
GFAP-β		More than 432	- Predominant isoform in human non-myelinating SCs	[7,9]
GFAP-γ		Less than 432	- Cells of the corpus callosum of the human brain - Brain, spleen, and bone marrow of mice	[8,10,11]
GFAP-δ/GFAP-ε		431	- Mainly in human astrocytes of the subventricular zone of the human	[11,15]
GFAP-κ		438	- Predominant isoform in human enteric glia cells - Human astrocytes - Mouse brain and spinal cord	[7,8,11]
GFAP-ζ		More than 432	- Human astrocytes	[11,12]
GFAP+1	GFAPΔEx6	347	- Human astrocytes - Long-processed astrocytes in Alzheimer’s disease and chronic epilepsy	[11,12]
	GFAPΔ164	366		[11,12]
	GFAPΔ135	374		[13]
	GFAPΔEx7	418		[9]

**Table 2:** Key studies focusing on the clinical significance of GFAP+ EVs.

Type of Biofluidic	Isolation Method	GFAP Detection Platform	Enrichment Strategy	Cohort Size	Clinical Association	Ref.
Blood plasma	ExoQuick exosome kit	ELISA	Sorption on streptavidine-agarose ultralink resin with GLAST biotinylated antibodies	12 pts. with early stage of AD vs. 10 matched cognitive normal controls	GFAP level in plasma ADEVs was significantly less in AD pts. than controls pts.	[37]
Blood plasma	ExoQuick exosome kit	Western blotting	No enrichment	12 pts. with schizophrenia vs. 12 controls	The significantly higher concentration of exosomal GFAP in the schizophrenia smpl. is suggestive of selective enrichment of exosome protein astrocytic origin only in the pts. samples. Exosomal samples from both groups were similar in the level of synaptophysin, suggestive of the presence of neuronal-derived exosomes irrespective of disease status.	[38]
Blood plasma	Differential centrifugation	High-sensitivity flow cytometry (individual detection)	No enrichment	Patients with SED (n = 31), MDD (n = 31), and healthy matched controls (n = 61)	Patients with SED had significantly higher concentrations of AQP4-positive and GFAP-positive EVs and EVs co-expressing AQP4/GFAP than patients with MDD and healthy controls.	[39]
Blood plasma	Differential centrifugation with ultracentrifugation and ultrafiltration	High-sensitivity flow cytometry (beads- based method)	Sorption of EVs on latex beads coated with antibodies to GFAP	Glioblastoma multiforme pts. with no tumor recurrence for over one year (n = 6) and after first relapse (n = 14)	In both groups, C5b-9 was predominantly detected on tumor-specific circulating EVs (GFAP+ EVs) with high VEGF-A expression, while C5b-9 was significantly less frequent on EVs with low VEGF-A expression. GFAP+VEGF+dimMMP2-C5b-9+ EVs were rarely detected in pts. without relapse, suggesting their potential utility as biomarkers for a favorable relapse-free prognosis. In recurrent pts., a positive correlation was observed between GFAP+VEGF+bright MMP2+C5b-9+ EVs and MGMT gene promoter methylation levels (r = 0.543; *p* < 0.05).	[43]
Blood serum	Differential centrifugation with ultracentrifugation	High-sensitivity flow cytometry (individual detection)	No enrichment strategy	8 pts with glioblastoma multiforme progressed early, late and without progression vs. 3 controls (non-cancers)	Pts. with glioblastoma have CD9+/GFAP+/Survivin+ and CD9+/Survivin+ exosomes that are released into the circulation and that early reductions in their numbers following anti-survivin immunotherapy might be associated with longer progression-free survival.	[44]
Blood plasma	Size-exclusion chromatography	Ultrasensitive single-molecule array	No enrichment	93 trauma patients (75 with TBI and 18 without TBI) were analyzed	EV-GFAP levels were significantly elevated in TBI patients compared with non-TBI trauma patients at admission and 15 h. A positive head CT was associated with 2.85 (95% CI: 1.18–6.91)-fold increased EV-GFAP, whereas EV-NfL and EV-T-Tau levels were not affected. None of the tested EV biomarkers were associated with 1-year mortality or 6–12 months’ functional outcome.	[41]
Blood serum	ExoQuick exosome kit	Ultrasensitive single-molecule array	No enrichment	72 TBI patients and 20 controls	EV GFAP concentrations were elevated in moderate and severe TBI compared with controls (*p* < 0.001) and could distinguish controls from moderate (AUC = 0.86) or severe TBI (AUC = 0.88). Increased EV GFAP and EV NfL levels were associated with lower 1-year Glasgow Outcome Scale–Extended scores (*p* < 0.05).	[42]
Blood plasma	ExoQuick ultra-EV kit	Western blotting	Exo-flow beads coated with GLAST biotinylated antibody	Plasma samples from 18 acute ischemic stroke pts. at 24 h (D1), 7 days (D7), and 30 days (D30) post-symptoms onset, and 9 healthy controls	Post-stroke ADEV GFAP levels were elevated at D1 and D7 but not D30 compared with controls (*p* = 0.007, *p* = 0.019, and *p* = 0.344, respectively). A positive correlation was observed between the modified Rankin scale at D7 and ADEV GFAP at D1 (r = 0.58; *p* = 0.010) and D7 (r = 0.57; *p* = 0.013), respectively.	[40]

Note: CT—computer tomography; AD—Alzheimer’s disease; GLAST—glutamine aspartate transporter, ADEVs—astrocyte-derived EVs; SED—stress-induced exhaustion disorder; TBI—traumatic brain injury; MMD—major depressive disorder; AQP4—aquaporin 4; NfL—neurofilament light chain; MMPs—matrix metalloproteinases.

**Table 3:** Overview of ExoCarta and Vesiclepedia entries reporting GFAP in EVs, including identification methods and critical assessment of evidence for vesicular and sub-vesicular localization.

Database	ID	Ref.	Sample Type/Source (Biological Origin)	Method	Localization of GFAP in EVs (Established/Not Established)	Summary of Localization Evidence
ExoCarta	GFAP (gene_id=2670; ExoCarta_2670), Experiment ID 224	[62]	Homo sapiens; neuroblastoma cells (SH-SY5Y)	Mass spectrometry; Western blotting	Not established	GFAP is reported as a “protein identified” within EVs based on MS; the study provides general physical/molecular EV characterization (electron microscopy and enrichment markers), but lacks GFAP-specific evidence (e.g., immunogold labeling and protease-based treatment).
ExoCarta	GFAP (gene_id=2670; ExoCarta_2670), Experiment ID 834–835	[63]	Homo sapiens; retinal pigment epithelial cells (ARPE-19)	Western blotting; mass spectrometry	Not established	The record confirms the presence of GFAP by MS and general EV validation (microscopy, NTA, and EV markers), but does not indicate its specific localization (intraluminal, surface-associated, or co-precipitated); GFAP is not investigated in localization-specific experiments.
ExoCarta	GFAP (gene_id=2670; ExoCarta_2670), Experiment ID 191	[64]	Homo sapiens; squamous-cell carcinoma cells (A431)	Mass spectrometry	Not established	The study emphasizes protein/exosome isolation and proteomic analysis of the secretome/100,000 g pellet; GFAP appears in the proteomic list, but no data are provided to support its vesicular or sub-vesicular topology.
ExoCarta	Gfap (gene_id=24387; ExoCarta_24387)	[65]	Rattus norvegicus; adipocytes/adipose tissue (OLETF rats)	Unspecified	Not established	The ExoCarta entry indicates the presence of GFAP in adipose tissue-derived EVs; however, the extracted record fragment does not specify the identification method for GFAP. Based on the PubMed annotation, this is EV proteomics using MS, which does not establish the intravesicular localization of GFAP.
Vesiclepedia	exp_id=354	[66]	Homo sapiens; plasma	Mass spectrometry [Orbitrap Velos]; Western blotting	Not established	Presence of GFAP is based on MS in the exosome fraction; the study compares proteomics across different isolation/stability conditions but does not provide GFAP-focused localization evidence (e.g., immunogold labeling or protease-based treatment).
Vesiclepedia	exp_id=590	[67]	Homo sapiens; colorectal cancer cells (e.g., HCT-15)	Mass spectrometry [LTQ]	Not established	High-throughput EV proteomics: GFAP is reported as an identified protein, corresponding to the level of “presence in an EV-enriched fraction,” without direct evidence of sub-vesicular localization.

Note: ID—unique identifier assigned to each record/object in the corresponding database.

## Data Availability

The data presented in this study are available upon request from the corresponding authors. The restriction of access does not hinder scientific transparency, but is aimed at protecting the copyrights of the authors of the article (figures).

## Abbreviations

The following abbreviations are used in this manuscript:

PC	Protein corona
EVs	Extracellular vesicles
GFAP	Glial fibrillary acidic protein
ADEVs	Astrocyte-derived EVs
CD9, CD63, TSG101, ALIX	Canonical EV markers
SCs	Schwann cells
SGCs	Satellite glial cells
SC-EVs	EVs from SCs
SGC-EVs	EVs from SGCs
DNP	Diabetic neuropathy
DINP	Drug-induced neuropathy
PTEN	A dual-substrate specificity phosphatase, a product of the *PTEN* gene, a negative regulator of the PI3K/AKT/mTOR signaling pathway
PNS	Peripheral nervous system
CNS	Central nervous system
BBB	Blood–brain barrier
DRG	Dorsal root ganglia of spinal nerves
NTA	Nanoparticle tracking analysis
MS	Mass spectrometry
NfL	Neurofilament light chain
STAT3, AP-1	Transcription factors, regulating GFAP expression
CT	Computer tomography
AD	Alzheimer’s disease
MMD	Major depressive disorder
GLAST	Glutamine aspartate transporter
SED	Stress-induced exhaustion disorder
AQP4	Aquaporin 4
MMPs	Matrix metalloproteinases
ELISA	Enzyme-linked immunosorbent assay
